# Subhepatic perforated acute appendicitis in a patient with midgut malrotation: A case report and review of the literature

**DOI:** 10.1016/j.ijscr.2022.107249

**Published:** 2022-05-28

**Authors:** Giuseppe Evola, Andrea Lanaia, Roberto Cantella, Cristina Di Fidio, Giovanni Francesco Di Fede, Luigi Piazza

**Affiliations:** a General and Emergency Surgery Department, Garibaldi Hospital, Piazza Santa Maria di Gesù 5, 95124 Catania, Italy; b Department of Diagnostic Radiology, Neuroradiology and Interventional Radiology, Garibaldi Hospital, Piazza Santa Maria di Gesù 5, 95124 Catania, Italy

**Keywords:** Subhepatic acute appendicitis, Midgut malrotation, Maldescent of the caecum, Abdominal pain, Emergency surgery, Case report

## Abstract

**Introduction and importance:**

Subhepatic acute appendicitis (SHAA) is a very rare cause of acute abdomen, developing in association with two types of congenital anomalies like as midgut malrotation (MM) and maldescent of the caecum. Preoperative diagnosis of SHAA is a challenge because of its rarity and atypical presentation. Imaging may be helpful for determining the correct diagnosis. Surgery represents the standard treatment of SHAA.

**Case presentation:**

A 25-year-old Caucasian male presented to the Emergency Department with a one-day history of right lower quadrant (RLQ) abdominal pain, nausea and vomiting. Physical examination revealed RLQ abdominal rebound tenderness with guarding. Laboratory tests reported high levels of C-reactive protein and neutrophilic leukocytosis. Abdominal contrast-enhanced computed tomography showed a SHAA with intraluminal appendicolith, fat infiltration and pelvic fluid collection in a patient with MM. The patient underwent laparoscopic appendectomy: a retrocaecal subhepatic phlegmonous and perforated appendicitis was sectioned and removed with drainage of pelvic abscess. The postoperative course of the patient was uneventful.

**Clinical discussion:**

SHAA is characterized by anatomical variation of appendix and atypical presentation. Preoperative clinical diagnosis of SHAA is very difficult and imaging may be helpful for determining the correct diagnosis, as well as confirming MM or maldescent of the caecum. Laparoscopic appendectomy represents the correct treatment of SHAA.

**Conclusion:**

SHAA is a rare surgical emergency that should be considered in the differential diagnosis of patients with RLQ abdominal pain. Preoperative diagnosis needs a high index of suspicion and is facilitated by imaging. Surgery represents the appropriate treatment of SHAA.

## Highlights

• Subhepatic acute appendicitis (SHAA) is a very rare cause of acute abdomen.
• SHAA develops in association with midgut malrotation and/or maldescent of the caecum
• Diagnosis of SHAA is generally a challenge because of the absence of specific clinical presentation.
• Abdominal contrast-enhanced computed tomography (CECT) is the best modality to identify SHAA if diagnosis is uncertain.
• Laparoscopic appendectomy is superior to open appendectomy in patients with atypical variants of the appendix.

## 1 Introduction

Acute appendicitis (AA) represents a common surgical emergency, accounting for 4–8% of all emergency department visits [1]. Diagnosis of AA is usually relative simple and is based on clinical symptoms, physical examination and radiology, however the malposition or anatomical variation of the appendix make it uncertain and can delay the surgical treatment favoring the chances of appendiceal rupture and the onset of complications such as abscess formation or peritonitis [2]. Subhepatic acute appendicitis (SHAA), characterized by anatomical variation of appendix and atypical presentation, develops in association with two types of congenital anomalies like as midgut malrotation (MM) [3] and maldescent of the caecum [4]. A rare case of SHAA in a patient with MM, managed in emergency by laparoscopic surgery, is presented with review of the literature in accordance with SCARE 2020 criteria [5]. The purpose of this case report is to remember that SHAA is a very rare cause of acute abdomen that may require emergency surgery.

## 2 Presentation of case

**Fig. 1:**
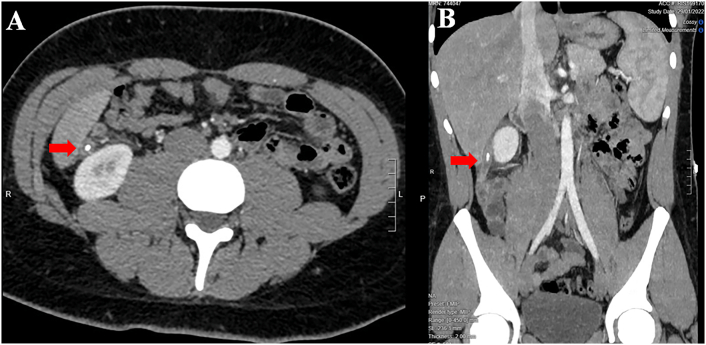
A,B. Abdominal CECT showing an inflamed subhepatic retrocaecal appendix with intraluminal appendicolith (red arrow). A transverse view, B coronal view. (For interpretation of the references to colour in this figure legend, the reader is referred to the web version of this article.)

**Fig. 2:**
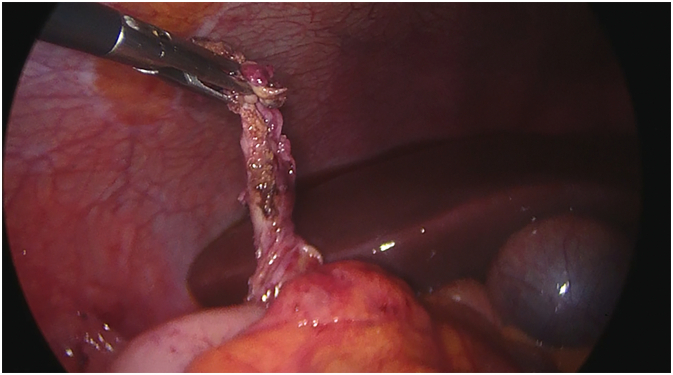
Subhepatic phlegmonous and perforated appendicitis: operative findings.

**Fig. 3:**
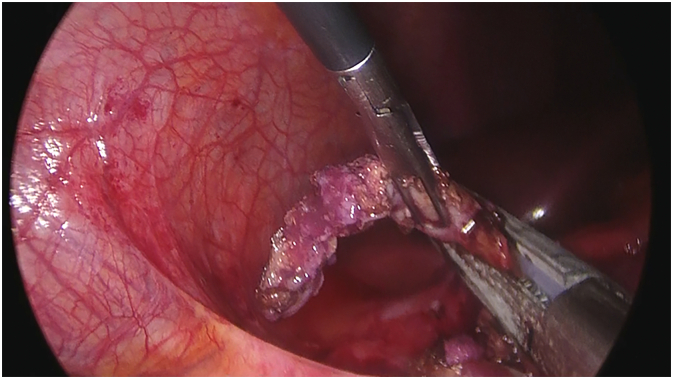
Section of the appendix with mechanical stapling device.

**Fig. 4:**
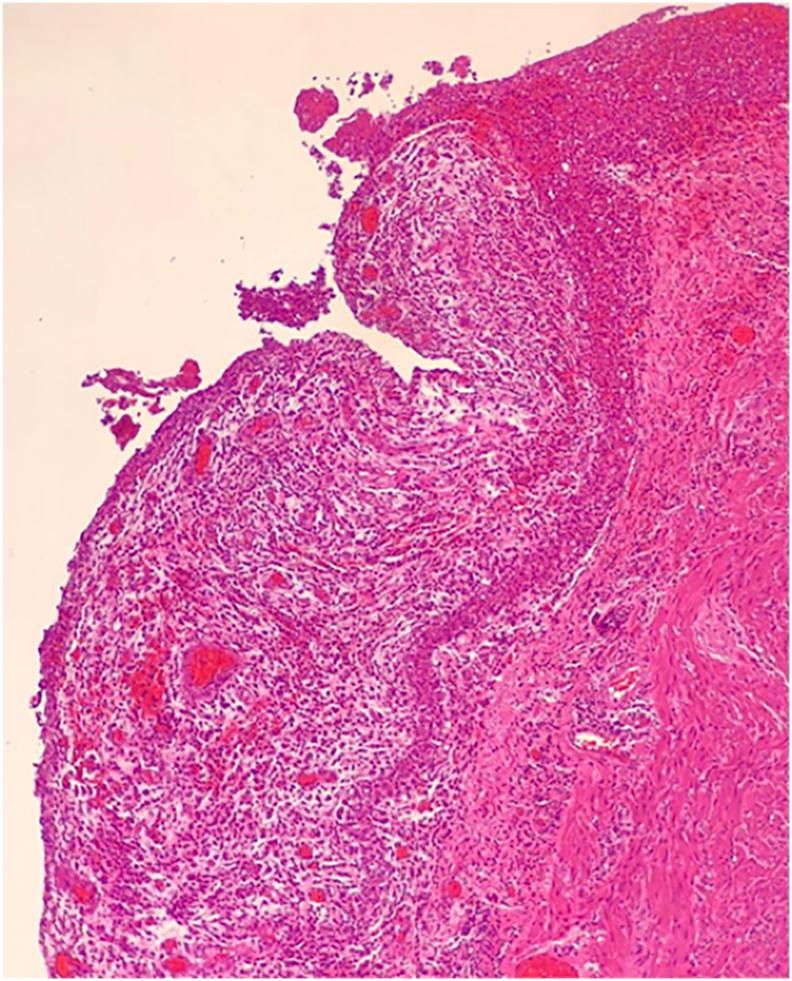
Photomicrograph section of perforated acute appendicitis (haematoxylin and eosin, original magnification x 10).

A 25-year-old Caucasian male presented to the Emergency Department with a one-day history of right lower quadrant (RLQ) abdominal pain, nausea and vomiting. He had no diarrhea and no fever, vital signs were normal. His past and familial medical histories were normal. He wasn't taking any drug, referred habit on smoking but denied alcohol consumption. The patient was employed by profession, married and of medium socio-economic status. Physical examination revealed mild abdominal distention, RLQ abdominal rebound tenderness with guarding and hypoactive bowel sound. Laboratory tests reported high levels of C-reactive protein (115.5 mg/L) and neutrophilic leukocytosis (WBC 15.600 10^3^/μL). The patient, presenting an Alvarado score of 7 (probable appendicitis), was initially managed with fluids, intravenous broad-spectrum antibiotics and bowel rest. After a negative echography for AA, abdominal contrast-enhanced computed tomography (CECT) revealed an inflamed subhepatic retrocaecal appendix with intraluminal appendicolith, fat infiltration and pelvic fluid collection (Fig. 1A,B). The patient, after understanding the severity of his medical condition and accepting surgery, was taken emergently to the operating room by experienced general surgeons for laparoscopic appendectomy under general anesthesia. After induction of pneumoperitoneum with the Veress needle and placement of two 12-mm trocars in the umbilical region and left iliac fossa and one 5-mm trocar in ipogastrium, we explored the peritoneal cavity with evidence of malrotated subhepatic caecum and pelvic abscess. We cut the lateral peritoneal reflection to medialize the caecum and the ascending colon with evidence of retrocaecal subhepatic phlegmonous and perforated AA (Fig. 2). In consideration of the patient's safety a fourth 5-mm trocar was inserted in the right iliac fossa to obtain a better medialization of the caecum. After dissection of mesoappendix and cauterization of appendicular artery with bipolar forceps, the appendix was sectioned using mechanical stapling device (Fig. 3) and removed in an endobag. After drainage of pelvic abscess, washing and aspiration of peritoneal cavity a pericaecal drain was placed. Patient was given an IV injection of Amoxicillin/Clavulanate 2 g twice daily and Metronidazole 500 mg thrice daily for five days. The postoperative course was uneventful: abdominal drain was removed on the 4th postoperative day, laboratory tests were unremarkable. The patient was discharged on the 5th postoperative day in a stable condition. Histopathological examination confirmed phlegmonous and perforated AA (Fig. 4). The patient tolered the advice provided and after a follow-up of six months is asymptomatic.

## 3 Discussion

This clinical case describes a rare SHAA causing acute abdomen in a patient with MM. AA is the most common abdominal surgical emergency in the world with an annual incidence of 96.5–100 cases per 100,000 adults [6]. SHAA accounts for 0.08% of all cases of AA and makes up an annual incidence of 0.09 per 100,000 adults [7]. Sub-hepatic position of caecum and appendix may occur in association with MM or arrested caecal descent. MM is caused by nonrotation or incomplete rotation of the primitive intestinal loop around the axis of superior mesenteric artery during weeks 5–10 of fetal life and subsequent abnormal fixation to the peritoneal wall. MM can manifest itself generally in the first month of life with bowel dysfunction and bilious vomiting, but in most of the cases it remained asymptomatic. The incidence of MM anomalies varies from 0.03% to 0.5% in the live births [8]. Arrested caecal descent occurs where the caecum lies in the subhepatic position but does not descend to the right iliac fossa. The *position* of the *appendix* is extremely variable: the most common location is retrocecal (74%) followed pelvic (21%), subcecal (1.5%), preileal (1%), and postileal (0.5%) [9]. The appendix can also show atypical locations such as subhepatic, left-sided [2], intraherniary [10], lateral pouch, mesocolic and lumbar [11]. The first case of SHAA, due to non-descent of caecum, was first described in 1955 by King [12]. Since then, only a few isolated cases of SHAA have been described in the literature with more reports of MM rather than nondescent of the caecum as a cause of this anatomical variant [13]. Diagnosis of AA is based on well-established clinical symptoms and signs, radiologic findings and surgeon experience. Knowledge of the variations in the position of the appendix is important because in AA its variable positions may produce variable symptoms and signs that mimic other diseases. Typical presentation (60% of cases) of AA begins with a vague abdominal discomfort around the epigastric region accompanied by nausea and vomiting; several hours later the pain migrates to the right iliac fossa near McBurney's point. Additionally fever, rebound tenderness, Rovsing's sign, psoas sign, diarrhea and anorexia may be observed. However one third of patients with AA complains abdominal pain in an unexpected location due to the various anatomical position of the appendix. Symptoms and signs of AA may be caused not only by the inflammation of the appendix but also by rare appendiceal neoplasms [14]. The main differential diagnosis of typical AA includes Crohn ileitis, mesenteric adenitis, right-sided colitis, intestinal perforation or obstruction [15], [16], incarcerated or strangulated hernia, regional enteritis, Meckel's diverticulitis, epiploic appendagitis, abdominal aortic aneurysm, mesenteric ischemia, renal colic, psoas abscess, testicular or ovarian torsion, ruptured ovarian cyst, ectopic pregnancy and pelvic inflammatory disease. SHAA, generally presenting with right upper abdominal pain, may be clinically indistinguishable from acute cholecystitis, liver abscess, perforated duodenal ulcer. This may lead to a delayed diagnosis of SHAA which can result in complications such as perforation of appendix, abscess formation, peritonitis and sepsis. However in our case report SHAA presented with *classic symptoms and* signs of AA [17]. Imaging may be helpful for determining the correct diagnosis of SHAA, as well as confirming MM or maldescent of the caecum. When abdominal ultrasound is the first radiological investigation, it has a high probability of misdiagnosis of SHAA. CECT is the best modality to identify SHAA with sensitivity of 88–100%, specificity of 92–98%, positive predictive value of 86–98%, negative predictive value of 95–100% [18]. MRI, compared to CT, has comparable sensitivity and specificity for the diagnosis of AA, but it is not universally available in emergency [19]. In our patient with RLQ abdominal pain and a negative echography for AA we conducted abdominal CECT to confirm the diagnosis: a SHAA was detected. Generally unusual location of the appendix may result in delay in appropriate diagnosis and treatment of AA [4], however in this case report there was no diagnostic delay. In many cases diagnosis of SHAA is made at laparoscopy especially if abdominal CECT is inconclusive. After establishing the diagnosis of SHAA, the surgical options are the same as for normal patients. Appendectomy represents the standard treatment of AA, although intravenous antibiotics may be considered first-line therapy in selected patients. After radiological diagnosis of SHAA we decided for laparoscopic appendectomy. In literature only few cases of laparoscopic approach in SHAA are reported. We believe that laparoscopic appendectomy in rare anatomical positions of appendix is a better option than the big incisions needed for adequate access and also it permits a better exploration of the abdominal cavity. Successful laparoscopic management requires a tailored approach altering standard port positions to allow optimal operative access [7]: the laparoscopic technique used in our case report was with four ports. AA is still one of the most common surgical emergencies with low morbidity and mortality if surgical treatment is not delayed. Morbidity and mortality increase if surgical treatment is delayed, often caused by misdiagnosis of AA. The mortality rate of AA is reported to be less than 1%, but it can be increased up to 5% in delayed diagnosed AA [20].

## 4 Conclusion

SHAA, occurring as result of MM and/or maldescent of the caecum, represents a rare surgical emergency that should be considered in the differential diagnosis of patients with right-sided abdominal pain. Its diagnosis is a challenge because of the absence of specific clinical presentation and needs a high index of suspicion. Imaging and/or laparoscopy are helpful in establishing the differential diagnosis of right-sided abdominal pain and in detecting SHAA as well as confirming MM or maldescent of the caecum. Laparoscopic appendectomy is the standard treatment of SHAA.

## Sources of funding

All the authors declare that this research didn't receive any specific grant from funding agencies in the public, commercial, or not-for-profit sectors.

## Ethical approval

Ethical approval has been exempted by our institution because this is a case report and no new studies or new techniques were carried out.

## Consent

Written informed consent was obtained from the patient, for publication of this case report and accompanying images. A copy of the written consent is available for review by the Editor-in-Chief of this journal on request.

## Registration of research studies

Not applicable.

## Guarantor

Giuseppe Evola.

## Provenance and peer review

Not commissioned, externally peer-reviewed.

## CRediT authorship contribution statement

Giuseppe Evola: Operated on the patient, drafting the manuscript, literature research.

Andrea Lanaia: Operated on the patient, drafting the manuscript.

Roberto Cantella: Drafting the manuscript, literature research.

Cristina Di Fidio: Drafting the manuscript, literature research.

Giovanni Francesco Di Fede: Drafting the manuscript, literature research.

Luigi Piazza: Revising the manuscript.

## Declaration of competing interest

All the authors certify that there is no conflict of interest regarding the material discussed in the manuscript.
